# The Effect of Various Plasma Gases on the Shear Bond Strength between Unfilled Polyetheretherketone (PEEK) and Veneering Composite Following Artificial Aging

**DOI:** 10.3390/ma12091447

**Published:** 2019-05-04

**Authors:** Mohamed Younis, Alexey Unkovskiy, Ashraf ElAyouti, Jürgen Geis-Gerstorfer, Sebastian Spintzyk

**Affiliations:** 1 Section Medical Materials Science & Technology, University Hospital Tuebingen, Osianderstrasse 2-8, 72076 Tuebingen, Germany; Geis-Gerstorfer@mwt-tuebingen.de (J.G.-G.); Sebastian.Spintzyk@med.uni-tuebingen.de (S.S.); 2 Department of Prosthodontics at the Centre of Dentistry, Oral Medicine, and Maxillofacial Surgery Dental School, Tuebingen University Hospital, Osianderstrasse 2-8, 72076 Tuebingen, Germany; Alexey.Unkovskiy@med.uni-tuebingen.de; 3 Department of Dental Surgery, Sechenov First Moscow State Medical University, Trubetskaya str. 8-2, 119991 Moscow, Russia; 4 Department of Conservative Dentistry, at the Centre of Dentistry, Oral Medicine and Maxillofacial Surgery Dental School, Tuebingen University Hospital, Osianderstrasse 2-8, 72076 Tuebingen, Germany; Ashraf.Elayouti@med.uni-tuebingen.de

**Keywords:** PEEK, plasma surface treatment, veneering composite resin, shear bond strength, thermocycling

## Abstract

This study investigated the effect of different gaseous plasma surface treatments on the shear bond strength between unfilled polyetheretherketone (PEEK) and veneering composite resin. The study followed ISO 10477 guidelines in preparing, bonding, and testing the samples. Specimens of unfilled PEEK were distributed to one of the following six surface treatment groups: reference, adhesive, argon, nitrogen, oxygen, and air plasmas. After milling, the specimens were wet polished using (P320) polishing discs. Bonding procedures were done according to the manufacturer’s instructions using (Opaquer + Dentine), except in the adhesive group (Visio.link + Opaquer + Dentine). Afterwards, thermal cycling for 5000 cycles between 5 and 55 °C in distilled water was conducted. Finally, the shear bond strengths of all groups were calculated, and mode of fracture was determined. Nitrogen surface treatment had the highest mean shear bond strength of 10.04 (±1.84) MPa, while the reference group showed the lowest value of 5.38 (±2.90) MPa. Regarding mode of fracture, all the specimens showed a 100% adhesive failure mode. Plasma surface treatment can be a reliable alternative method to the traditional protocol of bonding veneering composite resin to unfilled PEEK material.

## 1. Introduction

In the last few years, polyetheretherketone (PEEK) has become an attractive material for dental prostheses framework production due to its biocompatibility, light weight, and wear and chemical degradation resistance [1,2]. Despite these common advantages, the utilization of PEEK in the anterior esthetic zone is still challenging because of its opaque grey color [1]. This limitation can be overcome by the layering of PEEK frameworks with composite resins in order to achieve a better shade and translucency [3]. However, as PEEK surfaces are inherently inert and hydrophobic, the veneering composite resins suffer clinically from chipping, delamination, or fracture [4,5]. This has encouraged the investigation of various surface treatment techniques to achieve a superior adhesion between PEEK and veneering composites.

Traditionally, sandblasting and chemical etching are applied for surface treatment of different PEEK materials [6,7,8,9]. The utilization of plasma as an innovative approach to treating PEEK surfaces prior to bonding has also been used and has resulted in widely diverse results [10,11,12,13,14]. For instance, the studies of Stawarczyk et al. and Schmidlin et al. revealed no positive influence of helium plasma on the shear and tensile bond strength of ceramic-filled PEEK to two self-adhesive resin cements [12,15], whereas in the study of Zhou et al., the utilization of argon plasma improved the bond strength of PEEK to the tested resin cement and veneering composite [11]. Schwitalla et al. explored the influence of a cold, low pressure plasma mixture of argon/oxygen gases on the shear bond strength of three types of PEEK (unfilled PEEK, ceramic-filled PEEK, and pigment powder-filled PEEK) to veneering composite and observed insignificant improvement of the bond strength after combining sandblasting with plasma treatment [13]. Bötel et al. reported the positive impact of oxygen and argon–oxygen mixture plasmas on the shear bond strength of various PEEK types to three veneering composites [14].

Thus, only helium, argon, and oxygen plasma gases have been assessed for improving the bond strength between PEEK and veneering composite. Other gases, such as air and nitrogen, have not been tested before on PEEK materials despite the promising results that they have showed with other types of polymers. For instance, certain improvements in the adhesion of polyethylene polymers (PE) after air and nitrogen plasma treatment were revealed in the study of Lommatzsch et al. [16]. Such improvement in adhesion was also found in the study conducted by Noeske et al. on five different polymers after air plasma treatment and additionally the study conducted by Yavirach et al. on the adhesion between fiber-reinforced posts and a composite core material after nitrogen plasma [17,18].

Because different plasmas can be produced and controlled through application of various feeding gases [19]. The present study aimed to investigate the influence of plasma surface treatment with argon, oxygen, air, and nitrogen feeding gases on the shear bond strength between unfilled PEEK and veneering composite resin. The null hypothesis was that none of the applied feeding gases would be able to improve shear bond strength.

## 2. Materials and Methods

### 2.1. Specimens Design and Preparation

This study followed ISO 10477 guidelines for polymer-based crown and bridge materials. First, 120 specimens were milled of unfilled PEEK (Optima PEEK Juvora Ltd., Lancastershire, UK, LOT: J000009). The specimens’ dimensions were 20 mm × 10 mm × 2 mm. Each specimen was painted with black marker and polished in a polishing machine (Metaserv Motopol 12, Buehler UK LTD, Coventry, UK) with P320 polishing discs rotating at 150 RPM under constant water until complete disappearance of the painting. Afterwards, all specimens were cleaned in an ultrasonic bath (Sonorex, Bandelin, Berlin-Germany) containing 70% ethanol for 20 min and then left in air until they were completely dry.

To ensure standardization, surface roughness of eight specimens was measured using a Perthometer (Perthen, Mahr, Göttingen, Germany). The needle with a 2 μm diamond tip traversed the center of each specimen surface at a constant speed of 0.5 mm/s in an area of 3 mm length and width. Then, 121 measurement lines with 25 μm distance between the lines were performed. Average surface roughness (Ra) was calculated in analyzing software (MountainsMap Universal 7.2, Digital Surf, Besanco, France).

### 2.2. Surface Treatment and Bonding

Plasma surface treatment was done by using a Denta PLAS PC (Diener electronic GmbH, Ebhausen, Germany). The parameters of each surface treatment were adjusted to 10 min treatment duration, 0.3 mbar pressure, 20 °C temperature, 40 kHz frequency, and a power output of 100 W.

Four groups of 20 specimens each were treated with oxygen, nitrogen, argon, and air plasmas comprising n_oxygen_ = 20, n_nitrogen_ = 20, n_argon_ = 20, n_air_ = 20 groups. Then 20 more specimens were treated with Visio.link adhesive (Bredent; Senden, Germany,) and constituted the fifth group n_adhesive_ = 20. The remaining 20 specimens were left untreated and constituted the sixth group n_reference_ = 20.

**Figure 1:**
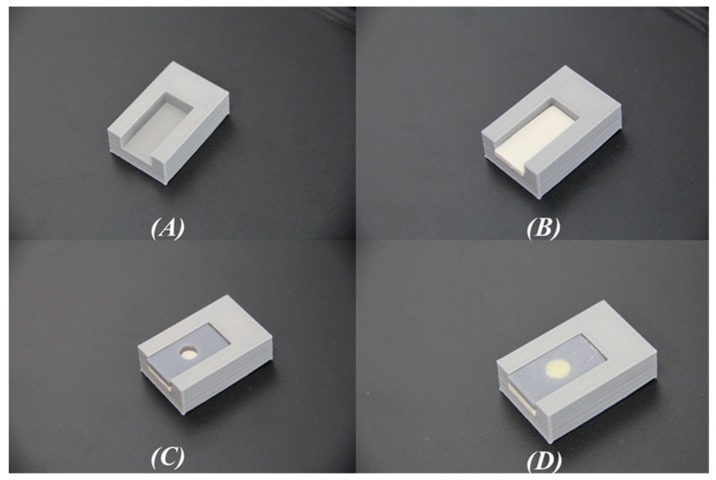
Pictures from (**A**–**D**) show the use of jigs during the process of bonding the veneering composite to the specimens.

Before bonding, bonding jigs were designed using a CAD software; Siemens NX 10.0 (Siemens PLM, Plano, TX, USA) for standardization of bonding procedures. It consisted of two parts; the first part was designed for the seating of PEEK specimens. The second part had of dimensions 20 mm × 10 mm × 2.5 mm, and bonding steps were made through a centralized hole with a diameter of 5 mm (Figure 1). 

**Figure 2:**
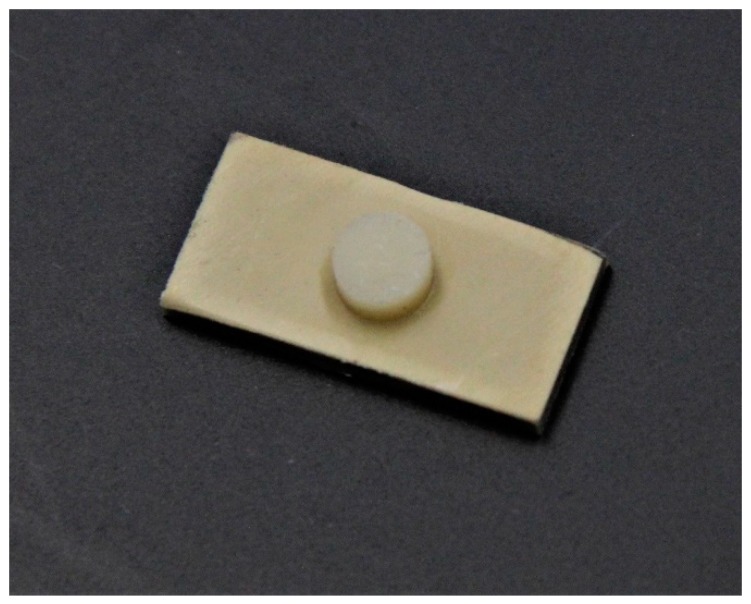
Finished specimen before testing.

Afterwards, bonding of veneering composite to PEEK was done following the manufacturer’s instructions by direct application of opaquer and A3 shade dentine composite paste (Sinfony veneering composite, 3M ESPE AG, Seefeld, Germany) (Figure 2).

Additionally, the surface area of the cured veneering composite in all specimens was captured under microscope Wild M400 photomacroscope (Wild Heerbrugg, Gais, Switzerland) with a DSLR camera (EOS 700D, Canon, Tokyo, Japan) and measured in Datinf software (Datinf GmbH, Tübingen, Germany). The veneered area was measured 3 times per sample, and the mean surface area was calculated.

### 2.3. Specimens Testing

Artificial aging for all specimens using a thermocycler (SD Mechatronik, Feldkirchen-Westerham, Germany) was done by immersion in distilled water for 5000 cycles at two temperatures 5 °C (±1) and 55 °C (±1) for 30 s in each with a dwell time of 20 s between the temperatures. Then, the fracture load was measured using a universal testing machine (Zwicki 1120, Zwick Ulm, Germany). A customized specimen holder was used to fix the specimens’ position throughout the testing procedure. A chisel-shaped rod applied force constantly parallel to the bond surface at a distance of 0.5 ± 0.02 mm from the surface of the PEEK specimen with 1 mm/min speed of crosshead and starting from a 0 N load, which increased gradually until fracture of the veneering composite occurred. Finally, the shear bond strength could be calculated according to the following equation:

(1) $$S=\frac{F}{A}$$

where *S* is the shear bond strength, *F* is the fracture load in Newtons, and *A* is the bonded area in mm^2^. Specimens that did not survive aging and showed premature debonding of veneering composites during thermocycling were assigned 0 MPa shear bond strength and considered as pre-failures.

### 2.4. Post Fracture Analysis

After shear bond testing, specimens were pictured again under microscope (Wild M400 photomacroscope, Wild Heerbrugg, Gais, Switzerland) to determine the type of failure. Three failure types were defined as follows:

1. Adhesive failure, which means no resin remnants were left on the PEEK surface.
2. Cohesive failure, where the failure was located in the bulk layer of the resin.
3. Mixed failure, where resin remnants were partially left on the PEEK surface and the PEEK surface was exposed.

### 2.5. Statistical Analysis

For statistical analysis, JMP 13.1 software package (SAS Corp., Heidelberg, Germany) was used. Means and standard deviations were used for descriptive analysis of roughness data, additionally a bar chart was used for shear bond strength results. Normal distribution was checked via a Shapiro–Wilk test. Afterwards, one-way ANOVA and post hoc Tukey tests were performed (α = 0.05).

## 3. Results

### 3.1. Surface Roughness

The results of surface roughness showed that polishing with P320 discs had an average roughness value of 1.01 µm (±0.1).

### 3.2. Shear Bond Strength

**Figure 3:**
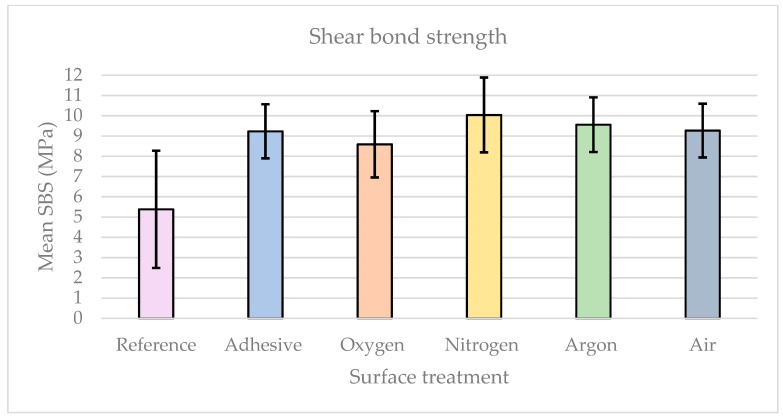
Bar chart of shear bond strength (SBS) results of study groups.

The results of the shear bond strength test revealed values of 5.38 MPa (±2.90), 9.23 MPa (±1.34), 8.59 MPa (±1.64), 10.04 MPa (±1.84), 9.56 MPa (±1.35), and 9.27 MPa (±1.33) for groups n_reference_ = 20, n_adhesive_ = 20, n_oxygen_ = 20, n_nitrogen_ = 20, n_argon_ = 20, and n_air_ = 20, respectively. The values of adhesive- and plasma-treated groups were significantly different from that of the reference group (*p* < 0.05), whereas no statistical difference was encountered between the adhesive and plasma groups (Figure 3). The null hypotheses that there is no influence of feeding gases on the bonding strength between PEEK and veneering composites had to be rejected.

### 3.3. Post Fracture Analysis

**Figure 4:**
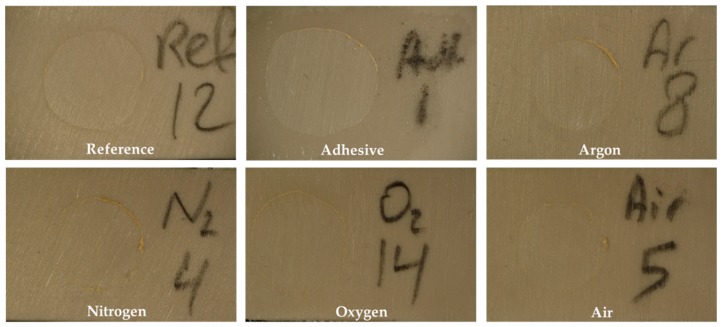
100% adhesive failures in all test groups.

All specimens showed 100% adhesive failure (Figure 4).

## 4. Discussion

Both the filled and unfilled types of PEEK were investigated in terms of bond strength to veneering composites or resin cements. In the literature, ceramic-filled PEEKs with different filler concentrations were the most used PEEK type [4,6,7,12,13,14,15,20,21,22], followed by unfilled PEEK [9,13,14,23,24,25,26]. Glass-filled PEEK, silica-filled PEEK, and carbon-filled PEEK gained less attention from researchers [11,26,27]. Various fillers and their percentages were reported to affect the bonding behavior to different veneering composites [13,14,28]. Unfilled PEEK was therefore used in this study to investigate the influence of surface treatment techniques on the bonding strength solely [13,14,26].

In the last decade, plasma technology was reported as an alternative to conventional sandblasting or chemical etching, because it overcomes the drawbacks of both. The potential effect of sandblasting is highly susceptible to the distance and angle of its application, pressure, grain size, and duration of treatment [25]. Therefore, this type of treatment is highly operator dependent and inconsistent. As PEEK is highly resistant to etching because of its chemical structure, its consistent surface treatment necessitates the application of highly powerful concentrated acids, such as sulfuric acid or piranha solution. Thus, chemical etching may cause injuries and is, for this reason, considered to be unsafe for dental clinic or laboratory settings [6,9,23,24]. In contrast, plasma treatment is environmentally friendly and can effortlessly treat complex-shaped structures and confine its effects to a superficial layer (10 nanometers depth) without altering the bulk properties of the treated material [29,30]. It delivers chemical functional groups that increase surface energy and wettability and can improve bonding to veneering resins [29,30,31]. On the other hand, there are lots of parameters that may influence plasma treatment processes, such as input power, pressure, temperature, and feeding gases [19]. Therefore, this study investigated the potential effect of various feeding gases on the bonding behavior of veneering composites to unfilled PEEK.

Prior to starting the plasma surface treatment, care was taken to standardize the surface roughness in all specimens. In the literature, the effect of surface roughness as a variable parameter on bonding strength between PEEK and veneering composite has not been well investigated yet, and the recommendations for the best values of surface roughness capable of producing the highest bond strength are still not clear. Rosentritt et al. revealed that the highest bonding strength values could be achieved after sandblasting with 50 μm alumina, which had an average roughness value of 0.96 μm (±0.07) [9]. For this reason, P320 silica carbide papers were used in the present study in order to bring the surface roughness to a comparable value of 1.01 µm (±0.1). Regarding the selection of a suitable adhesive agent, Visiolink was the chosen adhesive in this study, because it was well investigated and showed a positive effect on the bonding behavior of PEEKs and veneering composites in different studies [4,7,21,32]. 

The present study has assessed the influence of nitrogen, oxygen, argon, and air feeding gases on the bond strength between PEEK and veneering composites. Helium gas was not included in the study setup, as the findings of Stawarczyk et al. revealed no positive impact of helium plasma application [15], and the study of Schmidlin et al. proposed minor bond strength values after helium plasma surface treatment compared with conventional adhesive application [12]. In this study, all applied gases had a positive influence on the shear bond strength in comparison with the reference group and could reach bond strength values similar to that of the adhesive group. 

Zhou et al. reported a lower mean shear bond strength value of 5.4 MPa (±1.5) after argon plasma treatment in comparison with the mean value of 9.56 MPa (±1.35) obtained in the present study, although the treatment duration lasted longer (25 min) [11]. This difference could be attributed to the fact that higher polished silica-filled PEEK surfaces after treatment with 800 grit polishing discs were used before argon treatment in contrast with the unfilled PEEK and P320 polishing discs used in the present study.

The outcomes of the present study coincide with those from Bötel et al., as oxygen plasma treatment for 35 min improved the bonding of the veneering composite to unfilled PEEK significantly. The shear bond strength reached values up to 29.57 MPa (±3.71) in comparison with the mean value of 8.59 MPa (±1.64) obtained in this study. This difference in results may be attributed to the fact that in the study of Bötel et al., specimens were sandblasted before oxygen plasma treatment and Visio.link was applied afterwards, in addition to the fact that no artificial aging was performed [14].

As mentioned earlier, the literature lacks any reports on air and nitrogen plasma application for dental PEEK surface treatment. Nevertheless, the utilization of these feeding gases showed high bond strength in comparison with the control group. In non-dental literature, air plasma showed bond strength values in the range from 3.7 to 8.9 MPa, which is quite similar to those in our study (9.27 ± 1.33 MPa) [17]. The nitrogen plasma surface treatment yielded bond strength values around 20 MPa, which were higher than in our study 10.04 MPa (±1.84) [18]. It is important to note that both non-dental studies had different settings and measured bonding strength to different materials, which makes comparison to this study difficult. In the literature, many articles attributed the improvement of bond strength between tested materials mainly to formation of active carboxyl, hydroxyl, and amine functional groups, whose availability and intensity differ according to the type of gas used [18,19,33,34,35,36]. These functional groups can represent potential sites for chemical bonding between PEEK and veneering composites [13,14,19,37].

Concerning fracture mode analysis, the results of this study show adhesive failures, which was almost consistent with the study of Zhou et al. [11] and differed from the results of the study of Schwitalla et al. [13], in which an unfilled PEEK group treated with plasma showed 40% adhesive failures and 60% mixed failures. The reason could also be attributed to the variance between both study settings; in this study, bonding started directly without the application of an adhesive, and additional thermocycling was performed. In the study of Bötel et al., mixed failures dominated in the unfilled PEEK group, which could be attributed to the fact that all the specimens were sandblasted after polishing, and adhesive was applied in all plasma groups [14].

The limitations of this study were as follows: the full protocol for applying Sinfony veneering composite involves the application of a Rocatec sandblasting system; however, the objective of the study was to investigate the effect of plasma surface treatment solely. The effect of additional sandblasting followed by plasma treatment should be assessed in further studies. Further studies on this topic should address the utilization of various gas mixtures and perhaps a greater number of cycles after plasma treatment than 5000, as used in the present study according to the ISO 10477 guidelines.

## 5. Conclusions

The findings of this study suggest that plasma application alone can be a valid surface treatment method for providing optimal bond strength between unfilled PEEK and veneering composite resins. Thereby, the selection of feeding gases among nitrogen, oxygen, argon, and air is rather insignificant.
